# Histone deacetylase inhibitor givinostat has ameliorative effect in the colitis model

**DOI:** 10.1590/acb370503

**Published:** 2022-07-22

**Authors:** Cengiz Dibekoğlu, Oytun Erbaş

**Affiliations:** 1 MD. İstanbul Florence Nightingale Hospital – Department of General Surgery – İstanbul, Turkey.; 2 MD. Demiroğlu Bilim University – Faculty of Medicine – Department of Physiology – İstanbul, Turkey.

**Keywords:** Colitis, Tumor Necrosis Factor-Alpha, Rats

## Abstract

**Purpose:**

To investigate the effect of givinostat treatment in acetic acid-induced ulcerative colitis model in rats.

**Methods:**

Thirty male Wistar albino rats were used. Rats were randomly divided into three equal groups, and colitis was induced on 20 rats by rectal administration of %4 solutions of acetic acid. Twenty rats with colitis were randomly divided into two groups. %0.9 NaCl (saline) solution was administered intraperitoneally to the first group of rats (saline group, n=10) at the dose of 1 mL/kg/day. Givinostat was administered intraperitoneally to the second group rats (Givinostat group, n=10) at the dose of 5 mg/kg/day. Samples were collected for biochemical analysis. Colon was removed for histopathological and biochemical examinations.

**Results:**

Plasma tumor necrosis factor-α (TNF-α), pentraxin-3 (PTX-3), and malondialdehyde levels were significantly decreased in the givinostat group compared to the saline group (p<0.05, p<0.001, and p<0.001 respectively; p<0.001, p<0.001, and p<0.001, respectively). Colon TNF-α and prostaglandin F2 alpha (PGF-2) levels were significantly decreased (p<0.05, and p<0.001, respectively). The givinostat group had a significantly lower histologic score than saline group (p<0.001, and p<0.001, respectively).

**Conclusions:**

Givinostat, a good protector and regenerator of tissue and an anti-inflammatory agent, may be involved in the treatment of colitis in the future.

## Introduction

Ulcerative colitis is an inflammatory bowel disease, and its diagnosis is based on clinical, pathological, endoscopic, and radiological features^1^. Although the cause of the disease is not fully understood, some environmental genetic, and immunological factors are thought to play a role^2^. The main feature of ulcerative colitis is diffuse mucosal inflammation extending from the rectum to the proximal colon at varying levels^3^. Widespread superficial mucosal ulcerations develop with a complex mixture of inflammatory mediators resulting from severe inflammation. Histopathological features include the presence of a significant number of neutrophils within the lamina propria and the crypts, in which they form micro-abscesses^4^. Histological quantification of disease activity might be useful, as the histological severity of inflammation over time is a risk factor for colorectal neoplasia^5^. Mild cases show expansion of the lamina propria by lymphocytes and plasma cells, with infiltration of surface/crypt epithelium by neutrophils and/or crypt abscesses present in less than 50% of crypts. In moderately active cases, cryptitis and crypt abscesses account for more than 50% of the crypts. Severely active cases were defined by the presence of erosion or ulceration^6^^,^ 7.

The cytokine responses characterizing the inflammatory bowel diseases are the key pathophysiologic elements that govern the initiation, evolution, and, ultimately, the resolution of these forms of inflammation^8^. Levels of IL-1β, IL-4, IL-5, IL-6, IL-8, IL-10, IL-13, IL-33, IL-12p40, interferon gamma (IFNγ), and tumor necrosis factor-α (TNF-α) were significantly higher in patients with ulcerative colitis than in controls, suggesting a role for both cell-mediated and humoral immunity in the pathogenesis of ulcerative colitis. TNF-α upregulates other pro-inflammatory mediators such as IL-6 and IL-1β, thus amplifying the early sequences of the inflammatory cascade^9^^-^ 11.

Oxidative stress is another pathological factor that is associated with ulcerative colitis. Overproduction of reactive oxygen species (ROS) causes cell death by direct oxidative damage to cell proteins, membrane lipids, and DNA. Increased levels of ROS including superoxide, hydrogen peroxide, and peroxynitrite reduce the endogenous antioxidant levels that remove these toxic substances^12^^,^ 13. Another oxidative stress parameter is malondialdehyde (MDA), which is one of the end products of lipid peroxidation induced by ROS. Lipid peroxidation is a well-established mechanism of cellular injury in humans, and it is used as an indicator of oxidative stress in cells and tissues^14^. Measurement of MDA is widely used as an indicator of lipid peroxidation^15^.

The first treatment option to be used in the treatment of mild and moderate ulcerative colitis is 5-aminosalicylic acid (mesalazine), which includes oral and rectal mesalazine formulations and oral pro-drugs (sulfasalazine, olsalazine, and balsalazide)^3^^,^ 16, and, depending on the disease severity, oral prednisone, intravenous corticosteroids, ciclosporin, tacrolimus, and TNF-α antibody infliximab can be used.

Advantages of acetic acid-induced colonic injury are its low cost, ease of administration, widespread availability, and some resemblance to acute human intestinal inflammation. Thus, this easily inducible model may be useful in the initial screening of new drugs, evaluating mechanisms of mucosal healing, and determining the role of luminal factors in the perpetuation of nonspecific intestinal inflammation^17^.

Givinostat (ITF2357) is a novel, orally active histone deacetylase inhibitor (HDACi) that is in phase II clinical trials for the treatment of lymphoma^18^. Histone deacetylases (HDACs) have been implicated in the regulation of inflammation and attenuate cellular inflammation processes through inhibition of NF-jB activation and/or blockage of pro-inflammatory cytokine release^19^^,^ 20.

This study aimed to investigate the effect of givinostat treatment in acute ulcerative colitis model in rats. According to our hypothesis, givinostat in acute colitis will provide healing due to its anti-inflammatory properties. In the present study, we expected to create a successful colitis model and to increase the level of proinflammatory cytokines, development of oxidative stress and destruction of the colon tissue with the development of this colitis. We also expected regression of tissue and serum levels of proinflammatory cytokines, improvement of colitis in tissue examinations, and reduction of oxidative stress after givinostat treatment.

## Methods

The experimental protocol for all rats was approved by the Medical Ethics Committee for Experimental Animals (Science University Ethical number: 27211120). Experiments in our study were performed according to the guidelines in the Guide for the Care and Use of Laboratory Animals adopted by the National Institutes of Health. The rats used in the experiment were provided from the Experimental Animal Laboratory of our university.

In the present study, 30 male Wistar albino mature rats weighing between 200–250 g were used. Rats were housed in a temperature-controlled environment (22 ± 2°C) and under a 12-h light/12-h dark cycles. All animals were allowed free access to food and tap water.

### Experimental procedures

Rats were randomly divided into three groups, and acute colitis was performed on 20 rats. Ten untreated and orally fed rats were assigned as the normal control group, and they received no treatment. Acute colitis was induced by rectal administration of %4 solutions of acetic acid (AA) (Sigma Chemical Co). One mL of this solution was administered to every 20 rats. AA-induced experimental model of colitis was conducted according to the method previously established by Elson *et al*.^17^. After anesthesia with ether, a soft catheter was introduced 6 cm from the anus, and AA was slowly applied inside. Before the catheter was withdrawn, 1 mL of air was applied to disperse the AA well in the colon. During all these procedures, care was taken to avoid leakage from the anus. Then, 20 rats with colitis were randomly divided into two groups, %0.9 NaCl (saline) solution was administered intraperitoneally to the first group rats (saline group, n=10) at the dose of 1 mL/kg/day. Givinostat was administered intraperitoneally to the second group rats (givinostat group, n=10) at the dose of 5 mg/kg/day. All treatments were continued for 15 days. At the end of the study, all animals were euthanized, and blood samples were collected by cardiac puncture for biochemical analysis. The colon was removed for histopathological and biochemical examinations.

### Histopathological evaluation of colon

Formalin-fixed colon sections (4 μm) were stained with hematoxylin & eosin (H&E). All sections were photographed with an Olympus C-5050 digital camera mounted on an Olympus BX51 microscope.

Scores were assigned using the following criteria of MacPherson and Pfeiffer^21^ and modifications of this criteria by Fabia *et al*.^22^ and MacPherson and Pfeiffer^23^. Five scores from 0 to 4 were determined. In stage 0, the epithelium is intact and there are no leukocytes or hemorrhage. In stage 1, there are <25% disrupted epithelium, focal leukocyte infiltration, and focal hemorrhage. In state 2, 25% disrupted epithelium, focal leukocyte infiltration, and focal hemorrhage were defined. In stage 3, 50% disrupted epithelium, widespread leukocytes, and hemorrhage were seen. Stage 4 was with >50% disrupted epithelium, extensive leukocyte infiltration, and hemorrhage.

### Measurement of plasma TNF-α levels

Plasma TNF-α level was measured using commercially available enzyme-linked immunosorbent assay (ELISA) kit (Biosciences). The plasma samples were diluted 1:2, and TNF-α was determined in duplicate according to the manufacturer’s guide. The detection range for the TNF-α assay was *<*2 pg/mL.

### Evaluation of plasma pentraxin-3 levels

Plasma pentraxin-3 (PTX-3) levels were measured in each 100 μL sample by standard ELISA apparatus at 450 nm by using a PTX-3 kit (Uscn Life Science Inc., Wuhan, China). PTX-3 levels were determined in duplicate according to the manufacturer’s guide.

### Detection of TNF-α, Nrf-2, PGF-2 in the colon

The obtained frozen colon tissue was homogenized with a glass homogenizer in 1 mL of buffer containing 1 mmol/L of phenylmethylsulfonyl fluoride (PMSF), 1 mg/L of pepstatin A, 1 mg/L of aprotinin, and 1 mg/L of leupeptin in phosphate buffered saline (PBS) solution (pH 7.2), and then was centrifuged at 12,000 rpm for 20 minutes at 4°C. The supernatant was then collected, and the total protein was determined utilizing the Bradford method. The levels of TNF-α, prostaglandin F2 alpha (PGF-2), Nrf-2 in the tissue supernatants were measured using an ELISA kit specific for known rat TNF-α. The measurement of TNF-α, PGF-2, Nrf-2 were performed in a step-by-step fashion consistent with the protocol booklet of the ELISA kit. The cytokine contents in the colon tissue were expressed as pg/mg tissue.

### Measurement of plasma lipid peroxidation

Lipid peroxidation was determined in plasma samples by measuring MDA levels as thiobarbituric acid reactive substances (TBARS). Briefly, trichloroacetic acid and TBARS reagent were added to the plasma samples, then mixed and incubated at 100°C for 60 min. After cooling on ice, the samples were centrifuged at 3,000 rpm for 20 min, and the absorbance of the supernatant was read at 535 nm. MDA levels were expressed as nM, and tetraethoxypropane was used for calibration.

### Statistical analysis

Data analyses were performed using Statistical Package for the Social Sciences (SPSS), version 15.0, for Windows. The groups of parametric variables were compared by Student’s t-test and analysis of variance (ANOVA). The groups of nonparametric variables were compared by Mann-Whitney U test. Results were given as mean ± standard error of the mean (SEM). A value of p<0.05 was accepted as statistically significant, and P<0.001was accepted as statistically highly significant.

## Results

### Biochemical findings

Table 1 shows all biochemical plasma and colon tissue data. According to the results, plasma TNF-α, PTX-3 and MDF levels were significantly increased in the colitis + saline group compared to the normal control group (p<0.05, p<0.001, and p<0.001, respectively; p< 0.001, p<0.001, and p<0.001 respectively).

Similarly, colon TNF-α and PGF-2 levels were significantly increased in the saline group compared to the control group (p<0.05, and p<0.001, respectively). On the other hand, in the group treated with givinostat, these values were significantly lower than in the saline-treated group (p<0.05, and p<0.001, respectively).

On the colon Nrf-2 side, a significant decrease was detected in the saline group compared to the control group, and there was a significant increase in the givinostat-treated group compared to the saline group (p<0.001, and p<0.001, respectively).

**Table 1:** Biochemical results of plasma and colon tissue$.

Biochemical parameters	Control group	Colitis + saline	Colitis + givinostat
Plasma TNF-α (pg/mL)	20.13 ± 1.8	58.2 ± 4.4*	26.5 ± 3.1##
Plasma Pentraxin-3 (ng/mL)	0.8 ± 0.09	2.8 ± 0.3**	1.2 ± 0.1##
Plasma MDA (nM)	56.1 ± 2.7	136.1 ± 14.9**	85.3 ± 6.8##
Colon TNF-α (pg/mg tissue	87.3 ± 6.5	182.6 ± 11.05*	106.2 ± 5.3#
Colon Nrf-2 (pg/mg protein)	38.2 ± 2.2	13.9 ± 2.8**	25.1 ± 0.9##
Colon PGF-2 (pg/mg protein)	22.9 ± 1.7	1,210.7 ± 105.2**	344.6 ± 9.8##

$ Results were presented as mean ± standard error of the mean (SEM). Statistical analyses were performed by one-way analysis of variance;

* p<0.05

** p < 0.001 different from normal group;

# p<0.01

## p<0.001 different from colitis and saline group; TNF-α: tumor necrosis factor-α; MDA: malondialdehyde; PGF-2: prostaglandin F2 alpha.

### Histopathological score and findings

**Figure 1:**
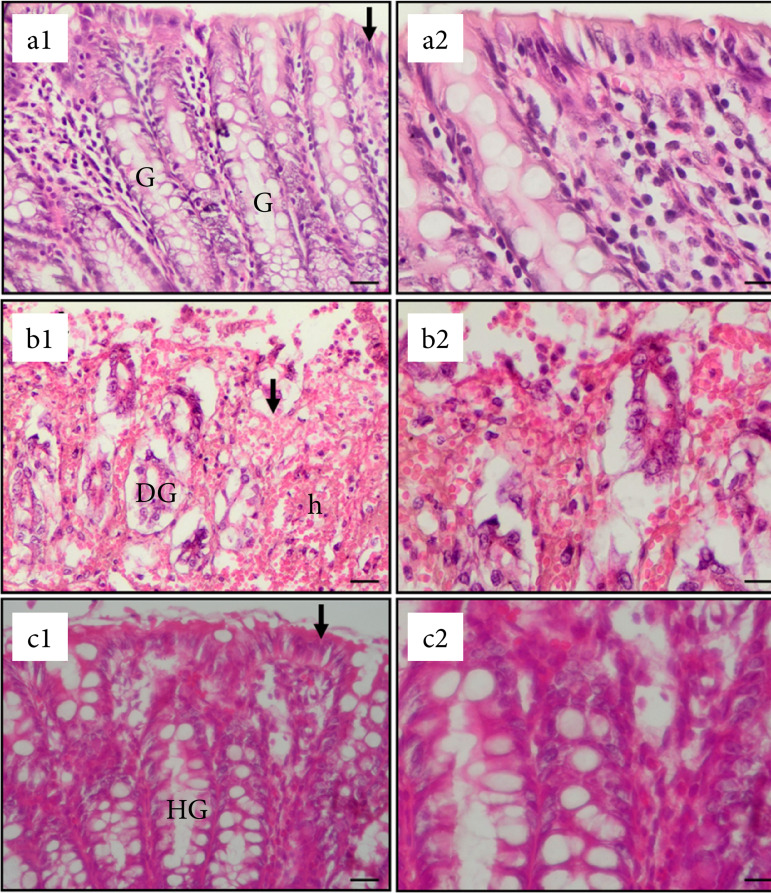
Microscopic images of colon sections of rats with colitis.

The curative effect of givinostat on colitis is visible on histopathological images of rats (Figs. 1a-1c). In Fig. 1b1-1b2, the damaged epithelium, degenerated glands, and bleeding belonging to the saline-treated colitis group are clearly noticeable compared to the normal control group in Figs. 1a1-1a2. In contrast, in Figs. 1c1-1c2, the marked improvement in the givinostat-treated group is clearly noticeable compared to the saline-treated colitis group.

As shown in Table 2, the histological score was significantly higher in the saline-treated colitis group compared to the normal group. On the other hand, the givinostat-treated colitis group had a significantly lower score than the saline treatment group (p<0.001 and p<0.001, respectively).

**Table 2:** Histopathological scores of groups$.

Control group	Colitis + saline	Colitis + givinostat
0.3 ± 0.1	3.2 ± 0.3*	1.1 ± 0.2#

$ Results were presented as mean ± standard error of the mean (SEM). Statistical analyses were performed by one-way analysis of variance;

* p<0.001 different from normal group;

# p<0.001 different from colitis and saline group.

## Discussion

In the present study, we investigated the effects of givinostat, a novel HDAC inhibitor, on intestinal inflammation in an ulcerative colitis model. In this study, we evaluated tissue healing and preservation histologically, the amount of proinflammatory cytokines in the blood and tissue, and the presence of oxidative stress in tissues and plasma due to injury and inflammation. We found that givinostat was effective in the healing of colitis and the prevention of proinflammatory cytokines, tissue destruction, and reduction of oxidative stress.

The normal colonic mucosa provides an efficient barrier against the potentially harmful conditions that normally exist in the intestinal lumen^24^. In ulcerative colitis, inflammation begins, and it reduces transepithelial resistance^25^. This reduced transepithelial resistance has been attributed to an increased rate of single-cell apoptosis and tight junction dysfunction. In severe cases, more widespread epithelial apoptosis produces extensive mucosal ulcerations, including necrosis^26^.

In our study, we observed that AA successfully induced colitis. AA-induced colitis is a well-established model similar to human ulcerative colitis^22^. The grading system previously used for AA colitis was used for histopathological scoring^21^^,^ 23.

In the present study, givinostat regressed this adequately formed colitis in a statistically highly significant manner. Histological results showed that the colonic mucosa was much better in the givinostat-treated group than in the saline-treated group (Fig. 1). These regenerative and protective effects may be attributed to increased secretion of transforming growth factor beta1 (TGF-β1) and interleukin 8, and also expression of the tight junction proteins claudin-1, claudin-2, and occludin^27^. Thus, the separation of cells from each other is prevented, ulcers cannot form, and the disease cannot progress. Some studies show that Nrf2 strengthens the intestinal epithelial structure. Occludin and zona occludens proteins increase in intestinal epithelial layers as a result of stimulation of the Nrf2 signal cascade^28^. In addition to its well-established anti-inflammatory properties, it may also perform tissue protection and healing through the above mechanisms.

There is solid evidence that HDACs are involved in different pathways of inflammation and intestinal inflammation^27^^,^ 29. A link between HDACs and intestinal inflammation has been demonstrated in some studies^27^^,^ 30.

Oxidative stress plays an important role in the development and progression of ulcerative colitis. With the progression of ulcerative colitis, activities of infiltrating leukocytes, neutrophils, and macrophages are greatly increased in the colon, resulting in increased generation of pro-oxidant molecules^12^. Cytokines-induced elevated myeloperoxidase (MPO) level also leads to ROS^13^. The enzymatic defense systems in the colonic mucosa are involved in maintaining the reduced state of proteins and protecting the cells against (ROS), drugs, and heavy metal ions^31^. Further, various cytokines and pro-oxidants activate the nuclear factor kappa B (NF-κB), which is a redox-sensitive transcription factor and plays an important role in the development and progression of ulcerative colitis. NF-κB is regulated by the redox state of cells^32^. It was revealed that also NF-κB increases, lipoxygenase (LOX), cyclooxygenase-2 (COX-2), and inducible nitric oxide synthase (i-NOS) gene expression, further increase oxidative stress^33^^-^ 35. Oxidative stress increases NF-kB release, resulting in increased Cox-2 gene transcription, triggering prostaglandin release that further promotes inflammation. In the present study, we measured the colon PGF-2 levels to determine the extent of inflammation. Colon PGF-2 secretion was found to be statistically significantly lower in the givinostat-treated colitis group compared to the saline-treated colitis group. In our opinion, this shows that givinostat inhibits COX-2 gene transcription by reducing oxidative stress and regressing inflammation.

Nuclear factor-erythroid 2 (NF-E2)-related factor 2 (Nrf2), a redox-sensitive transcription factor, which is essential for protection against oxidative/xenobiotic stresses, has been known to attenuate inflammation. It is another key regulator of cellular response to inflammatory cytokines and oxidative stress in multiple tissues and cell types. Nrf2 pathway is regarded as the most important in the cell to protect against oxidative stress^36^. When Nrf2 is exposed to inflammation or oxidative stress, it phosphorylates and translocates into the nucleus, giving rise to the transcription of proteins and antioxidant enzymes. Nrf2 deficiency results in increased inflammation and oxidative stress-induced tissue damage^37^^,^ 38.

In the present study, the colon Nrf2 level was measured. It was seen that the Nrf2 level in the group given givinostat was found to be significantly higher than the group that was not given givinostat. Plasma MDA levels were also measured to indicate the degree of oxidative stress. MDA levels were found to be significantly lower in the group given givinostat than in the group that not received it. MDA is an end product of lipid peroxidation induced by ROS. It is a reliable marker of lipid peroxidation, which is a well-established mechanism of cellular injury and used as an indicator of oxidative stress^39^. All these findings predicted that the administration of givinostat significantly reduces oxidative stress in the AA-induced colitis model as seen in plasma MDA and colon tissue Nrf2 levels.

The inflammatory response affects the formation of ulcerative colitis and increases the production of proinflammatory cytokines such as TNF-α, IL-1β, IL-6, and IFN-γ^11^^,^ 40. Some promote inflammation and are called pro-inflammatory cytokines, whereas other cytokines suppress the activity of proinflammatory cytokines and are called anti-inflammatory cytokines. For example, IL-4, IL-10, TGF-β, and IL-37 are potent anti-inflammatory cytokines, and they have the ability to suppress genes for proinflammatory cytokines such as IL-1, TNF, and the chemokines^11^^,^ 41.

TNF-α is a pro-inflammatory mediator that has been shown to play an integral role in the pathogenesis of inflammatory bowel disease (IBD)^9^^,^ 40. Transcription of the TNF-α gene in activated monocytes, macrophages, platelets, adipocytes, and T cells results in the secretion of TNF-α. Circulating soluble TNF-α binds to TNF-α receptors mediate multiple biologic effects, including activation of other macrophages, further augmentation of the T cell response, expression of adhesion molecules by vascular endothelium, recruitment of neutrophils to local sites of inflammation, stimulation of edema, inactivation of coagulation, and induction of granuloma formation^9^. Nuclear factor kappa beta (NF-κβ) is a pivotal transcription factor that increases the expression of many cytokines, enzymes, and adhesion molecules. TNF-α also prolongs inflammation by activating NF-κβ-dependent pathways, which contributes to ulceration and degradation of the mucosa through the release of matrix metalloproteinases (MMP)^42^. TNF-α upregulates other pro-inflammatory mediators such as IL-6 and IL-1β, thus amplifying the early sequences of the inflammatory cascade^9^^,^ 11. In this study, we determined TNF levels in both plasma and colon tissue. Both colon and plasma TNF levels were statistically significantly lower in the givinostat-treated group than in the saline-treated group. Many studies have shown that HDAC inhibitors can prevent inflammation and colitis. These studies have shown that the effect is mostly caused by the prevention of pro-inflammatory mediators (TNF-α and IL-1β) and oxidative stress^27^^,^ 29 ^,^ 30 ^,^ 43 ^,^ 44.

PTX-3 is an acute-phase protein representing the long pentraxin subfamily^44^. Production of PTX-3 is strongly induced by cytokines like interleukin 1, TNF-α, and by toll-like receptor (TLR) agonists, but not by interleukin 6 (IL-6) or interferons^45^^,^ 46.

PTX-3 is expressed in various cells, such as dendritic cells, monocytes, endothelial cells, and neutrophils during inflammatory processes^43^. Although PTX-3 belongs to the same family as C-reactive protein (CRP), it is released more rapidly during inflammation than CRP. In addition, CRP is produced by the liver, but PTX-3 is released directly from neutrophils^46^. PTX-3 is considered a good marker of inflammation. In our study, PTX-3 levels were found to be statistically significantly lower in the givinostat treatment group than in the saline group. The low PTX-3 level indicates that the inflammation was significantly reduced in the group treated with givinostat.

## Conclusions

The findings of present study indicate that givinostat may be effective in the prevention and treatment of colitis. As our study shows, it achieves this effect by preventing the release of important pro-inflammatory cytokines, reducing oxidative stress, and preventing the increase in the permeability of the colon wall in ulcerative colitis. The primary goal in the treatment of ulcerative colitis is to provide mucosal healing. Therefore, givinostat, which can provide this, may be involved in the treatment of this disease in the future. There will be a need for both experimental and clinical studies by more researchers on this subject in the future.
